# Tyrosine 842 in the activation loop is required for full transformation by the oncogenic mutant FLT3-ITD

**DOI:** 10.1007/s00018-017-2494-0

**Published:** 2017-03-07

**Authors:** Julhash U. Kazi, Rohit A. Chougule, Tianfeng Li, Xianwei Su, Sausan A. Moharram, Kaja Rupar, Alissa Marhäll, Mohiuddin Gazi, Jianmin Sun, Hui Zhao, Lars Rönnstrand

**Affiliations:** 1 Division of Translational Cancer Research, Department of Laboratory Medicine, Lund University, Medicon Village, Lund, Sweden; 2 Lund Stem Cell Center, Department of Laboratory Medicine, Lund University, Lund, Sweden; 3 School of Biomedical Sciences, Faculty of Medicine, The Chinese University of Hong Kong, Shatin, Hong Kong; 4 Department of Surgery, Faculty of Medicine, The Chinese University of Hong Kong, Shatin, Hong Kong; 5 Department of Pathogen Biology and Immunology, School of Basic Medical Sciences, Ningxia Medical University, Yinchuan, People’s Republic of China; 6 Department of Oncology, Skåne University Hospital, Lund, Sweden

**Keywords:** FLT3, FLT3-ITD, Activation loop, Acute myeloid leukemia, SHP2, Transformation, Survival, Microarray

## Abstract

The type III receptor tyrosine kinase FLT3 is frequently mutated in acute myeloid leukemia. Oncogenic FLT3 mutants display constitutive activity leading to aberrant cell proliferation and survival. Phosphorylation on several critical tyrosine residues is known to be essential for FLT3 signaling. Among these tyrosine residues, Y842 is located in the so-called activation loop. The position of this tyrosine residue is well conserved in all receptor tyrosine kinases. It has been reported that phosphorylation of the activation loop tyrosine is critical for catalytic activity for some but not all receptor tyrosine kinases. The role of Y842 residue in FLT3 signaling has not yet been studied. In this report, we show that Y842 is not important for FLT3 activation or ubiquitination but plays a critical role in regulating signaling downstream of the receptor as well as controlling receptor stability. We found that mutation of Y842 in the FLT3-ITD oncogenic mutant background reduced cell viability and increased apoptosis. Furthermore, the introduction of the Y842 mutation in the FLT3-ITD background led to a dramatic reduction in in vitro colony forming capacity. Additionally, mice injected with cells expressing FLT3-ITD/Y842F displayed a significant delay in tumor formation, compared to FLT3-ITD expressing cells. Microarray analysis comparing gene expression regulated by FLT3-ITD versus FLT3-ITD/Y842F demonstrated that mutation of Y842 causes suppression of anti-apoptotic genes. Furthermore, we showed that cells expressing FLT3-ITD/Y842F display impaired activity of the RAS/ERK pathway due to reduced interaction between FLT3 and SHP2 leading to reduced SHP2 activation. Thus, we suggest that Y842 is critical for FLT3-mediated RAS/ERK signaling and cellular transformation.

**Electronic supplementary material:**

The online version of this article (doi:10.1007/s00018-017-2494-0) contains supplementary material, which is available to authorized users.

## Introduction

Acute myeloid leukemia (AML) is a heterogeneous hematopoietic disorder. The molecular genetics of AML has been thoroughly investigated identifying loss-of-function mutations in transcription factors and gain-of-function mutations in receptor tyrosine kinases. The most commonly mutated genes include *NPM1, CEBPA, TET2, IDH, DNMT3A* and *FLT3*. Mutations in the receptor tyrosine kinase FLT3 occurs in more than 30% of AML patients [1]. The internal tandem duplication (ITD) is a repetition of sequence that encodes the juxtamembrane domain and is the most common oncogenic mutations in FLT3 that correlates with a poor prognosis [2]. Other oncogenic mutations include point mutations or ITD mutations in the kinase domain.

FLT3 belongs to the type III receptor tyrosine kinase (RTK) family that includes five members PDGFRA, PDGFRB, KIT, CSF1R and FLT3. The characteristic feature of this family is an extracellular domain consisting of five immunoglobulin-like domains and an intracellular kinase domain interrupted by a kinase insert. The extracellular domain mediates association of the dimeric ligand and thereby induces dimerization of the receptor. Dimerization of receptor initiates a tyrosine phosphorylation program resulting in phosphorylation of several tyrosine residues in the receptor which are essential both for receptor activation and signal propagation [3]. Activation of FLT3 subsequently activates downstream signaling cascades including PI3K-AKT and RAS-ERK signaling through various SH2 domain-containing proteins such as GAB2, SHC and SHP2 [3]. Although wild-type FLT3 requires its ligand FLT3 ligand (FL) for activation, oncogenic mutants are constitutively active. Oncogenic FLT3 binds essentially to the same docking molecules as wild-type FLT3, and therefore controls similar signaling pathways [4].

The *FLT3-ITD* mutations significantly increase the risk of relapse, and therefore limit disease-free and overall survival [2, 5]. Inhibition of FLT3 displayed promising results in clinical trials [6]. However, in most of the cases responses were not sufficient for treatment of AML with a single drug [6]. Inhibitors mostly reduce peripheral blood blasts transiently, and bone marrow responses are rare [7, 8]. Limited response to the inhibitors is mainly due to primary and secondary mutations in FLT3 that make the receptor resistant to the inhibitor [9]. The second-generation FLT3 inhibitor, AC220 (quizartinib), induced a composite complete remission rates of 44–54% which is much better than that observed with other FLT3 inhibitors. However, later studies indicate that treatment with this drug also suffers from problems of acquired secondary resistance [10]. A recent study suggests that the multi-kinase inhibitor midostaurin prolongs survival when used in combination with chemotherapy [11]. Thus, we still need a better understanding of the best way of targeting FLT3 for AML treatment.

Phosphorylation of the tyrosine residue in the activation loop is known to be the hallmark of activation of many tyrosine kinases. For example, phosphorylation of activation loop tyrosine residues of fibroblast growth factor receptor leads to a 500 to 1000-fold increase in substrate phosphorylation [12], and is also crucial for activation of the insulin receptor [13] and hepatocyte growth factor receptor (MET) [14]. However, in both KIT and the PDGFR activation of the receptors intrinsic kinase activity was independent of phosphorylation of the activation loop tyrosine residue [15–17]. In this report, we show that the activation loop tyrosine is critical for FLT3-induced downstream ERK1/2 signaling as well as for FLT3-ITD-mediated oncogenesis.

## Materials and methods

### Reagents, plasmids and antibodies

Human recombinant FLT3 ligand was from ORF genetics (Kópavogur, Iceland). The transfection reagent Lipofectamine 2000 was from Thermo Scientific and cycloheximide was from Sigma-Aldrich. pcDNA3-FLT3-WT, pMSCVpuro-FLT3-WT and pMSCVpuro-FLT3-ITD were described previously [18]. pMSCVpuro-FLT3-WT/Y842F and pMSCVpuro-FLT3-ITD/Y842F plasmids were generated by site-directed mutagenesis using QuikChange mutagenesis XL kit (Agilent Technologies). The anti-FLT3 antibody was a rabbit polyclonal antibody produced in-house. Mouse monoclonal anti-beta-actin, horseradish peroxidase-conjugated anti-FLAG antibody and mouse monoclonal anti-FLAG antibodies were from Sigma-Aldrich. Mouse anti-phosphotyrosine (4G10) antibody and mouse mono-ubiquitin antibody were from Millipore and Covance Research Products, respectively. Rabbit anti-ERK2, rabbit anti-phospho ERK1/2 (pThr202/pThr204), goat anti-AKT antibodies were from Santa Cruz Biotechnology. Rabbit anti-tubulin, rabbit anti-phospho-AKT (pSer473) rabbit anti-phospho GAB2 and rabbit anti-phospho-SHP2 were from Cell Signaling Technology.

### Cell culture, transient and stable transfection

COS-1 and 32D cells were obtained from Deutsche Sammlung von Mikroorganismen und Zellen (DSMZ). COS-1 cells were maintained in Dulbecco’s modified Eagle’s medium supplemented with 10% fetal bovine serum (FBS), 100 µg/ml streptomycin and 100 units/ml penicillin. 32D cells were cultured in RPMI 1640 medium containing 10% heat-inactivated fetal bovine serum (FBS), 100 µg/ml streptomycin and 100 units/ml penicillin. Transient transfection of COS-1 cells and stable transfection of 32D cells were described previously [19]. Transfected 32D cells were maintained in the IL3-containing medium as described earlier [20].

### Immunoprecipitation and western blotting

COS-1 cells were washed with cold PBS after 100 ng/ml ligand stimulation and lysed with Triton-X 100-based lysis buffer. 32D cells were starved of cytokines and serum for 4 h before stimulation. After stimulation cells were washed with cold PBS before lysis. Each ml of cell lysates was immunoprecipitated with 1 µg antibody and then processed for SDS-PAGE and western blotting analysis using the standard protocol [21].

### Cell viability, apoptosis and colony formation assays

32D cells were washed three times to remove cytokines and re-suspended in RPMI 1640 containing 10% FBS. PrestoBlue cell viability assay and apoptosis assay were described previously [22]. Colony formation assay was performed as described elsewhere [23].

### Animal experiment

Five male BALB/c nude mice in each group were used for animal experiments following Hong Kong animal ethical regulations. Mice were injected subcutaneously with 1,000,000 cells in 100 µl (1:1) PBS and Matrigel mixture. Mice were monitored for weight change and tumor size.

### Gene expression analysis using microarray

32D cells expressing FLT3-ITD or FLT3-ITD/Y842F were washed three times to remove cytokines and serum. Cells were starved in medium containing 0.5% serum overnight before extraction of total RNA using the RNeasy mini kit (Qiagen). Bio-analyzer was used to check the quality of RNA. Gene expression was analyzed using Affymetrix GeneChip Mouse Gene 2.0 ST arrays. Raw data were normalized using RMA normalization.

### Statistical analysis

All in vitro experiments were performed at least three times. Student’s *t* test and one-way ANOVA with Bonferroni’s post-tests were used for statistical analysis using GraphPad prism 5.0. Data were expressed as the mean ± SE and two-way *t* test was used.

## Results

### Expression of the FLT3/Y842F mutant results in reduced cell proliferation and enhanced apoptosis in myeloid cells

**Fig. 1:**
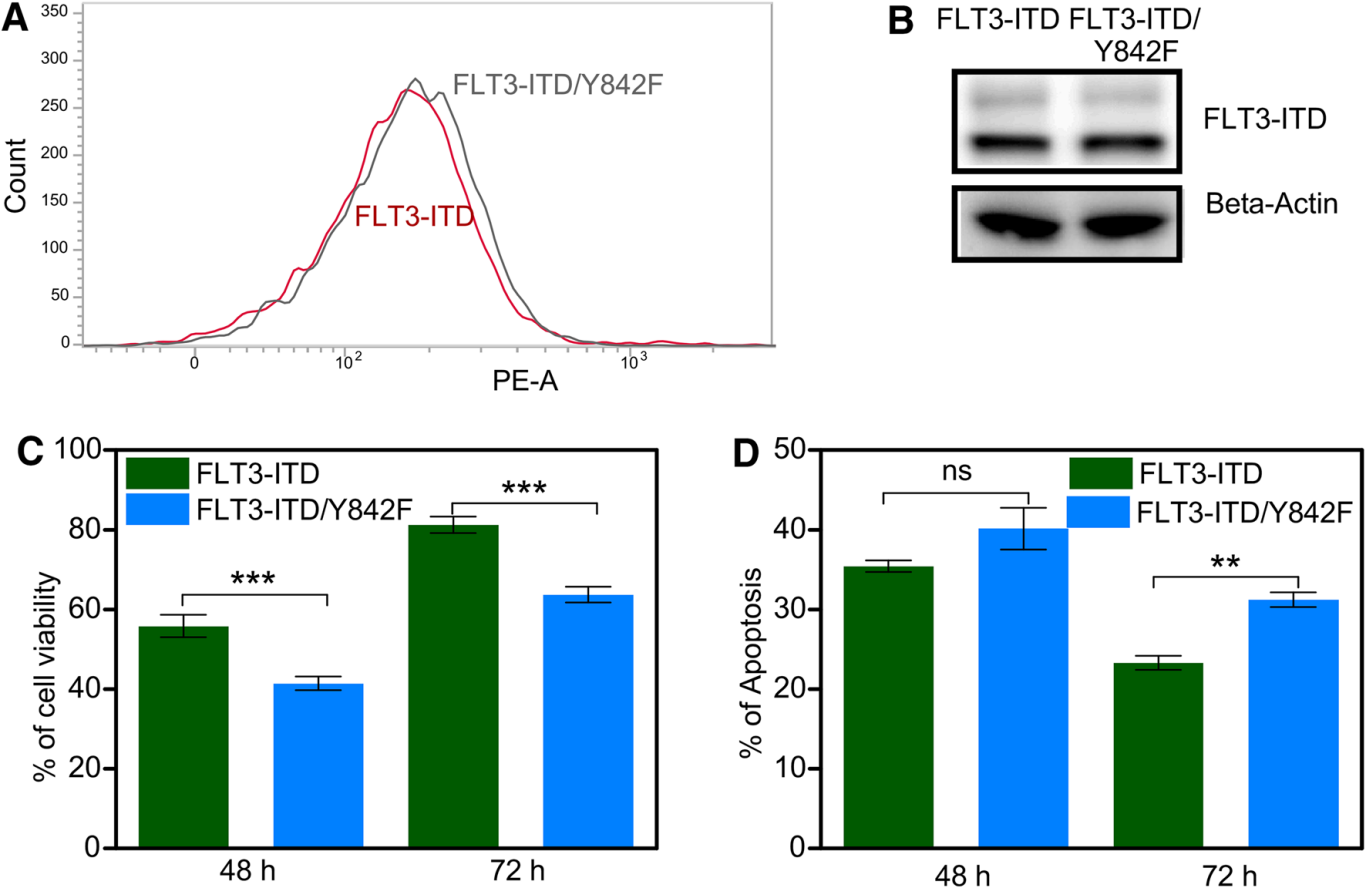
Y842F mutation reduces cell viability and increases apoptosis. **a** Cell surface expressions of FLT3-ITD and FLT3-ITD/Y842F in stably transfected 32D cells were analyzed by flow cytometry using PE-conjugated anti-FLT3 antibody. **b** 32D cells expressing FLT3-ITD and FLT3-ITD/Y842F were lysed and lysates were analyzed using SDS-PAGE and western blotting. **c** Cell viability was measured upon cytokine depletion using PrestoBlue cell viability assay after 48 and 72 h. **d** Apoptosis was measured after 48 and 72 of cytokine depletion using Annexin V and 7AAD kit. ***p < 0.001; **p < 0.01; *ns* not significant; *error bar* represents SEM

We have recently demonstrated that the activation loop tyrosine is of importance for KIT-mediated mitogenic signaling and KIT/D816V-mediated oncogenic transformation [15, 16]. Since FLT3 belongs to the same family of receptor tyrosine kinases as KIT, we hypothesized that the analogous FLT3 mutant would display a similar phenotype. We transduced a myeloid cell line, 32D lacking endogenous FLT3 expression, with oncogenic FLT3-ITD as well as with an activation loop tyrosine-to-phenylalanine mutant in ITD background, FLT3-ITD/Y842F. Since 32D cells are cytokine-dependent, withdrawal of cytokines leads to complete growth inhibition as well as to cell death. Both transfected cell lines displayed same cell surface expression of FLT3 (Fig. 1a, S1A and S1B) as well as total expression of FLT3 (Fig. 1b). We observed that while FLT3-ITD could fully support the viability of cytokine-starved 32D cells, FLT3-ITD-Y842F expressing cells displayed reduced viability. This suggests that, similar to KIT, phosphorylation of the FLT3 activation loop tyrosine is required for maintenance of cell viability (Fig. 1c). We also observed a significant increase in apoptosis in cytokine-starved cells expressing the FLT3-ITD/Y842F mutant compared to FLT3-ITD expressing cells (Fig. 1d).

### Cells expressing the Y842F mutant have an impaired capacity to form colonies in vitro and tumors in vivo

**Fig. 2:**
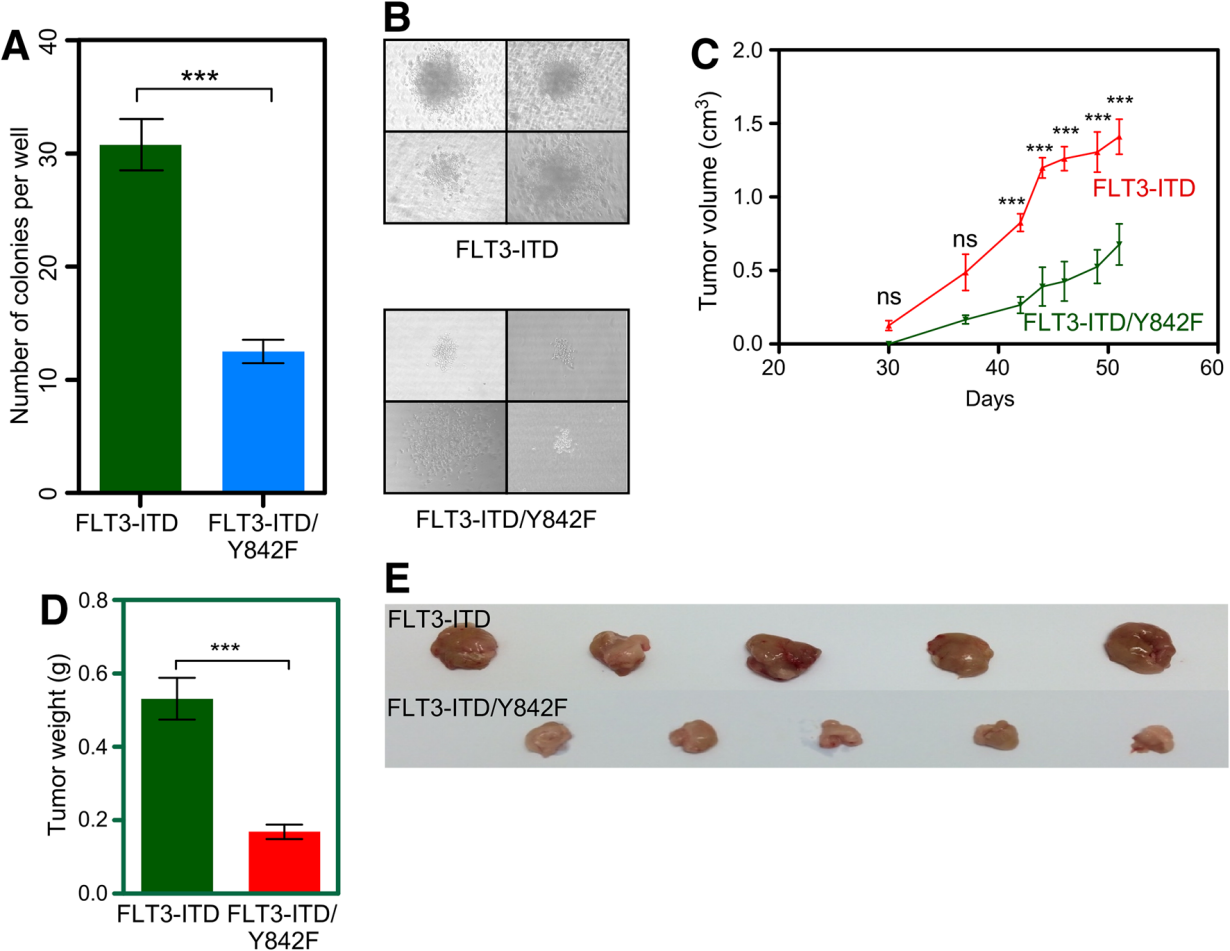
Y842F mutant has impaired colony formation and tumor formation capacity. **a, b** Cells were washed to remove cytokine and serum and seeded in methylcellulose medium. Colonies were counted 7 days after seeding. **c**–**e** Immunocompromised mice were injected subcutaneously with 32D cells expressing FLT3-ITD or FLT3-ITD-Y842F. Tumor volume was measured twice a week and tumor weight was measured after killing mice. ***p < 0.001; *ns* not significant; *error bar* represents SEM

As we observed that the FLT3-ITD/Y842F mutant cells had reduced cell viability and higher levels of apoptosis, we checked for their ability of in vitro colony formation in a semi-solid medium. We observed that cells expressing FLT3-ITD/Y842F induced a significantly lower number of colonies (Fig. 2a) as well as a reduced colony size (Fig. 2b) suggesting that the Y842F mutation reduces the transformation potential of FLT3-ITD. To verify our in vitro data in an animal model, we generated a mouse xenograft model by injecting 32D cells subcutaneously. The FLT3-ITD/Y842F mutant displayed significantly delayed tumor formation in xenotransplanted mice (Fig. 2c). Average tumor weight was reduced by 70% in mice injected with cells expressing FLT3-ITD-Y842F (Fig. 2d, e) compared to mice injected with FLT3-ITD expressing cells, suggesting that the phosphorylation of the activation loop tyrosine is an important event in FLT3-ITD-mediated transformation.

### Y842F mutation leads to downregulation of oncogenic signaling

**Fig. 3:**
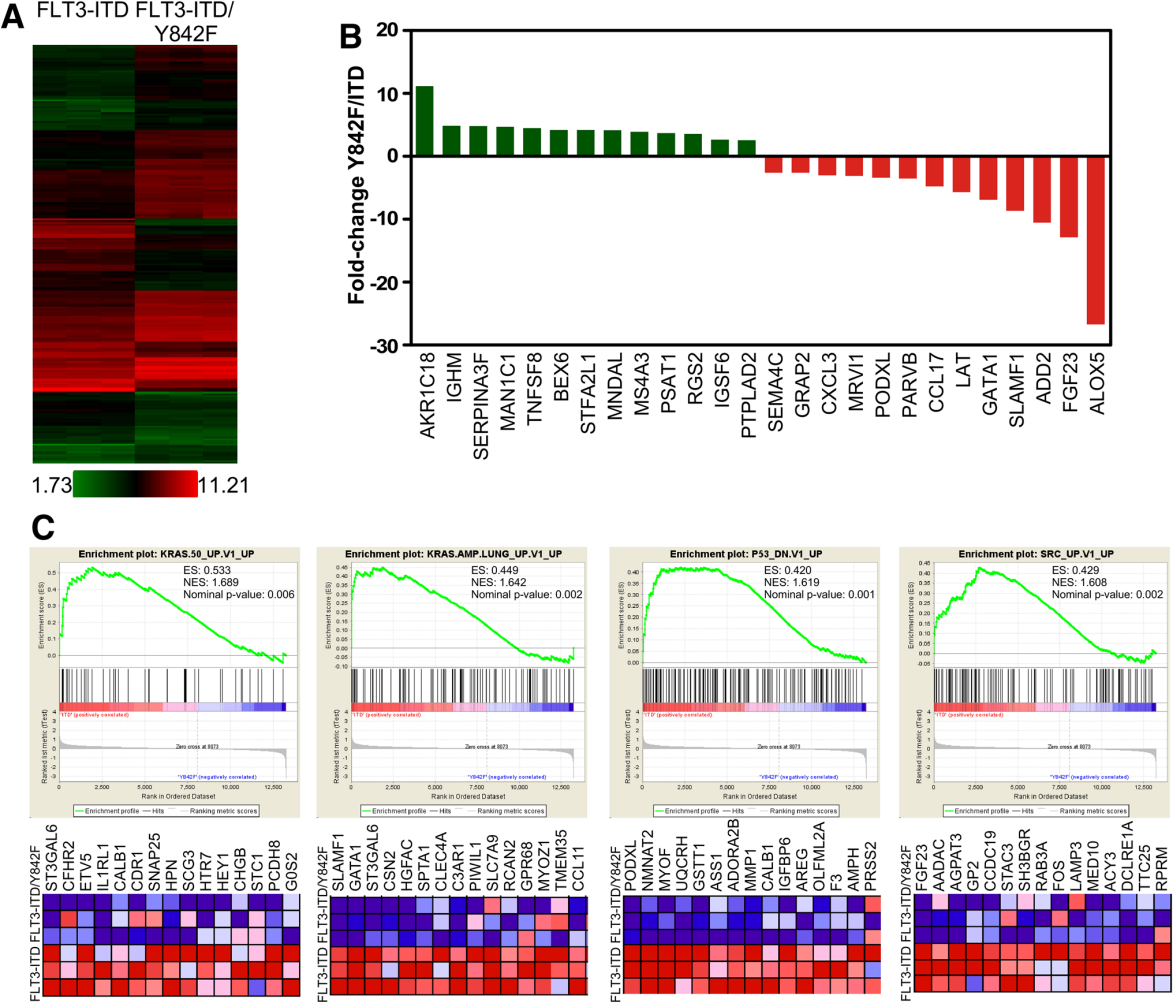
Y842F mutation changes FLT3-ITD-induced gene expression. **a** Heat map shows the difference in gene expression in between FLT3-ITD and FLT3-ITD-Y842F. **b** Upregulated and downregulated genes in Y842F mutant. **c** Gene set enrichment analysis between FLT3-ITD and FLT3-ITD-Y842F transfected cells shows enrichment of different oncogenic signatures

As we observed that the Y842F mutation diminishes FLT3-ITD-mediated cell viability, colony formation and tumor formation, we hypothesized that this mutation might influence FLT3-ITD-induced gene expression. Thus, we checked global gene expression using Affymetrix Mouse Gene 2.0 ST arrays (EMBL-EBI arrayexpress accession: E-MTAB-5258). Cells expressing FLT3-ITD or FLT3-ITD/Y842F display difference in gene expression patterns (Fig. 3a) indicating that Y842F mutation influences FLT3-ITD-mediated gene expression. Expression of anti-apoptotic genes and oncogenes was suppressed in cells expressing the Y842F mutant (Fig. 3b). We then used the gene set which were downregulated in cells expressing Y842F mutant and analyzed for gene ontology using DAVID Functional Annotation Bioinformatics Microarray Analysis (https://david.ncifcrf.gov). Result showed an enrichment of GO:0070374 (positive regulation of ERK1 and ERK2 cascade, *p* = 3.405*E*−08). Moreover, gene set enrichment analysis (GSEA) suggests that the deregulated genes are involved in several oncogenic pathways such as KRAS, SRC and loss of p53 (Fig. 3c). Since oncogenic signature genes were downregulated in Y842F expressing cells, it suggests that FLT3-ITD/Y842F has an impaired oncogenic capacity.

### Y842F mutation selectively inhibited FL-induced ERK1/2 activation

**Fig. 4:**
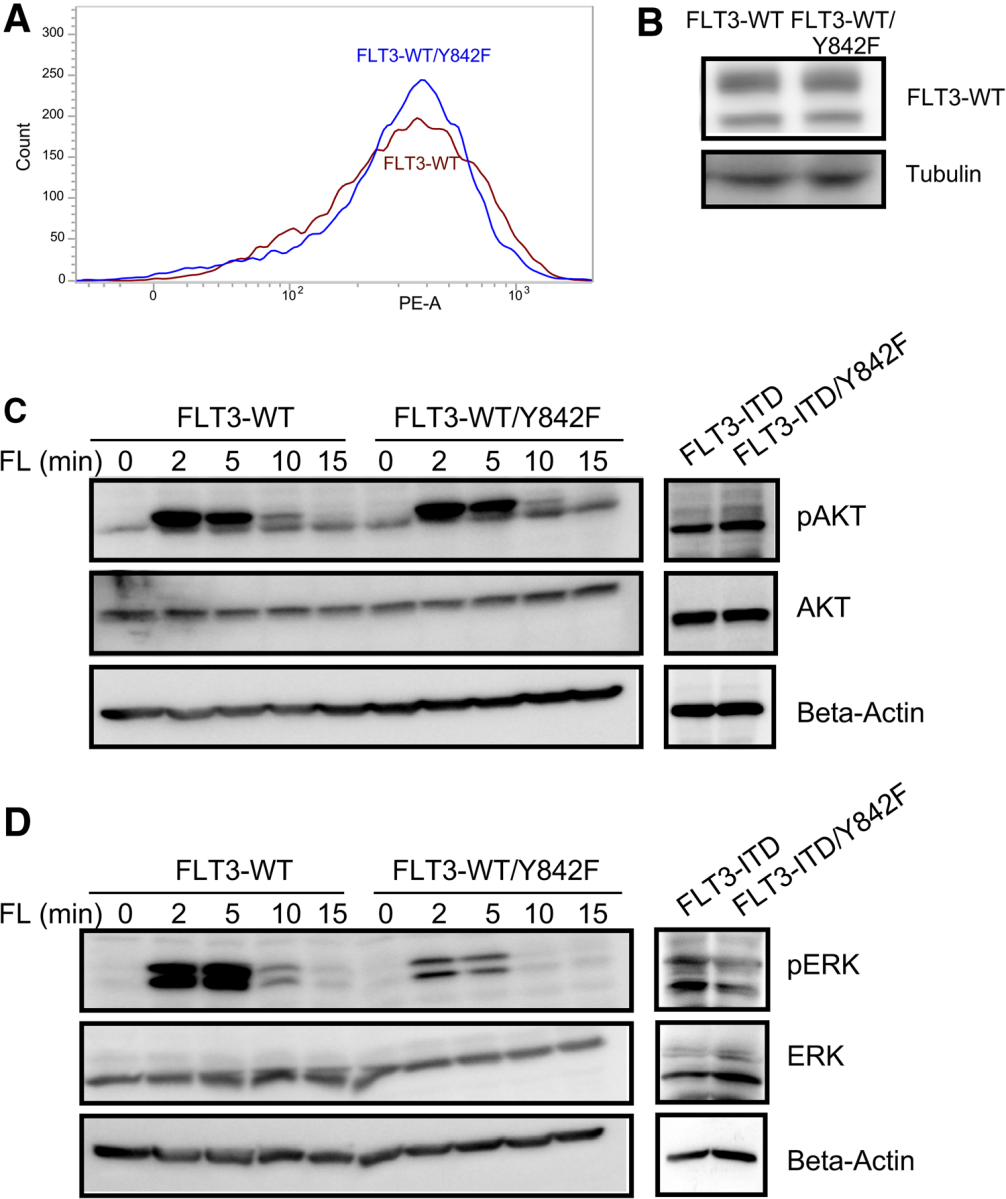
Y842F mutation selectively reduces ERK phosphorylation. **a** Cell surface expressions of FLT3-WT and FLT3-WT/Y842F in stably transfected 32D cells were analyzed by flow cytometry using PE-conjugated anti-FLT3 antibody. **b** 32D cells expressing FLT3-WT and FLT3-WT/Y842F were lysed and lysates were analyzed using SDS-PAGE and western blotting. **c, d** Cells were serum and cytokine starved for 4 h before stimulating with 100 ng/ml FL for different time points. Cells were then lysed and lysates were used for SDS-PAGE and western blotting analysis using anti-phospho-AKT (**c**) and anti-phospho-ERK1/2 (**d**) antibodies

To understand how mutation at Y842 affects FLT3-induced normal biological outcomes, we generated 32D cell lines expressing FLT3-WT and FLT3-WT/Y842F. Both cell lines expressed equal levels of FLT3 on the cell surface (Fig. 4a, S1C and S1D) as well as total FLT3 (Fig. 4b). Although expression of FLT3-ITD can partially support the growth of 32D cells upon cytokine withdrawal, cells expressing wild-type FLT3 cannot support the cell survival even when supplemented with FL. We analyzed FLT3 downstream signaling following stimulation with FLT3 ligand using phospho-specific antibodies. We observed that ligand stimulation of cells expressing the Y842F mutant activated phosphorylation of AKT to an equal extent as wild-type FLT3 (Fig. 4c). In contrast, mutation of Y842F led to a dramatic reduction in ERK1/2 phosphorylation (Fig. 4d). Similarly, FLT3-ITD-mediated constitutive activation of ERK1/2, but not AKT, was partially blocked by the Y842F mutation. However, we have not seen any reduction of STAT5 phosphorylation in cells expressing Y842F mutant (Fig. S2).

### FLT3-Y842F mutant displays impaired SHP2 activation

**Fig. 5:**
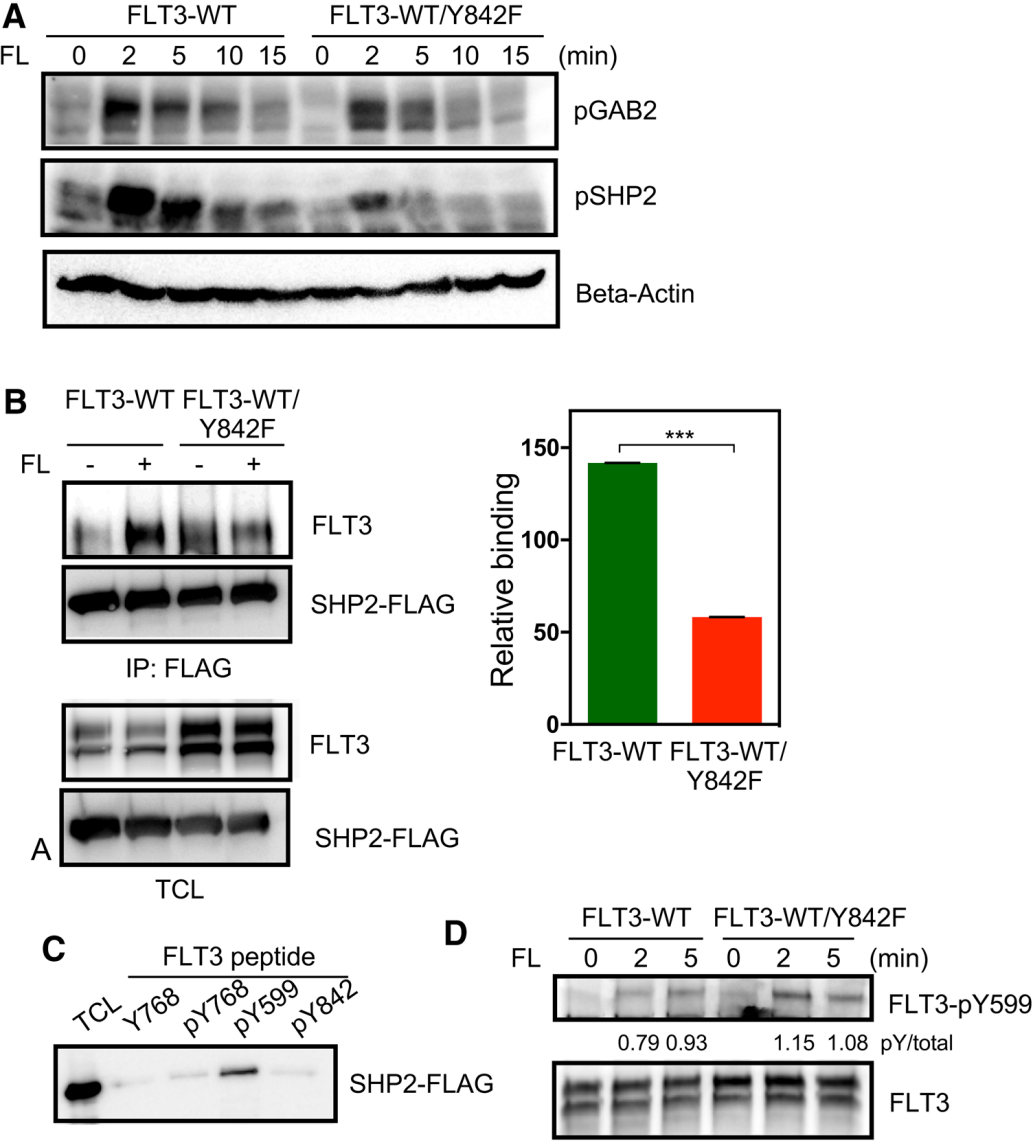
Y842F mutation reduces SHP2 phosphorylation. **a** Cells were serum- and cytokine-starved for 4 h before stimulating with 100 ng/ml FL for different time points. Cells were then lysed and lysates were used for SDS-PAGE and western blotting analysis using anti-phospho-GAB2 and anti-phospho-SHP2 antibodies. **b** COS-1 cells were transfected with FLAG-tag SHP2 and FLT3-WT or FLT3-WT/Y842F. One day after transfection cells were stimulated with 100 ng/ml FL for 5 min before lysis. Lysates were immunoprecipitated using 1 µg anti-FLAG antibody. ***p < 0.001; *error bar* represents SEM. **c** Phospho-peptides corresponding to the different FLT3 phosphorylation sites were coupled to Ultralink beads. Slurry of immobilized peptides was incubated with cell lysates from SHP2 expressing cells. **d** Cells were serum-starved for 4 h before stimulation followed by lysis. Lysates were used for immunoprecipitation followed by western blotting analysis

FLT3 induces ERK1/2 activation through multiple signaling cascades. The two key signaling molecules, GAB2 and SHP2, are involved in this pathway [3]. Therefore, we examined the levels GAB2 and SHP2 phosphorylation following FL stimulation. We observed that while the presence or absence of the Y842F mutation did not affect GAB2 phosphorylation, the phosphorylation of SHP2 was strongly decreased in cells expressing the Y842F mutant (Fig. 5a). This observation suggests that somehow phosphorylation of Y842 in the activation loop is required for FLT3-mediated SHP2 phosphorylation which in turn is involved in activation of ERK1/2 signaling. We have previously demonstrated that SHP2 associated with phosphotyrosine Y599 in FLT3 [24]. Since we did not see a difference in phosphorylation of GAB2 (which in other systems has been demonstrated to associate with SHP2) in the Y842F mutant, it is likely that Y842 is an important SHP2 association site in FLT3. We observed that while wild-type FLT3 was able to associate with SHP2, the ligand-activated Y842F mutant displayed reduced SHP2 interaction (Fig. 5b) suggesting that Y842 is one of the binding sites for SHP2 in FLT3, either directly or indirectly. To determine whether SHP2 directly associates with pY842, we used a synthetic phospho-peptide corresponding to the FLT3-Y842 site. We did not observe any association between SHP2 and the FLT3-pY842 peptide suggesting that pY842 is not a direct binding site for SHP2 (Fig. 5c). However, we cannot exclude the possibility that an adapter protein bridges the binding of SHP2 to FLT3. Another possible explanation of the reduced binding capacity of the FLT3-Y842F mutant is that it could be due to a reduction in FLT3-Y599 phosphorylation. Therefore, we compared FLT3-Y599 phosphorylation between wild-type and Y842F mutant. We did not observe any reduction of FLT3-Y599 site phosphorylation in Y842F expressing cells suggesting that the activation loop tyrosine has no role in regulating FLT3-Y599 phosphorylation (Fig. 5d).

### Mutation of the Y842 residue has no effect on ubiquitination or phosphorylation of FLT3 but leads to reduced stability of the protein

**Fig. 6:**
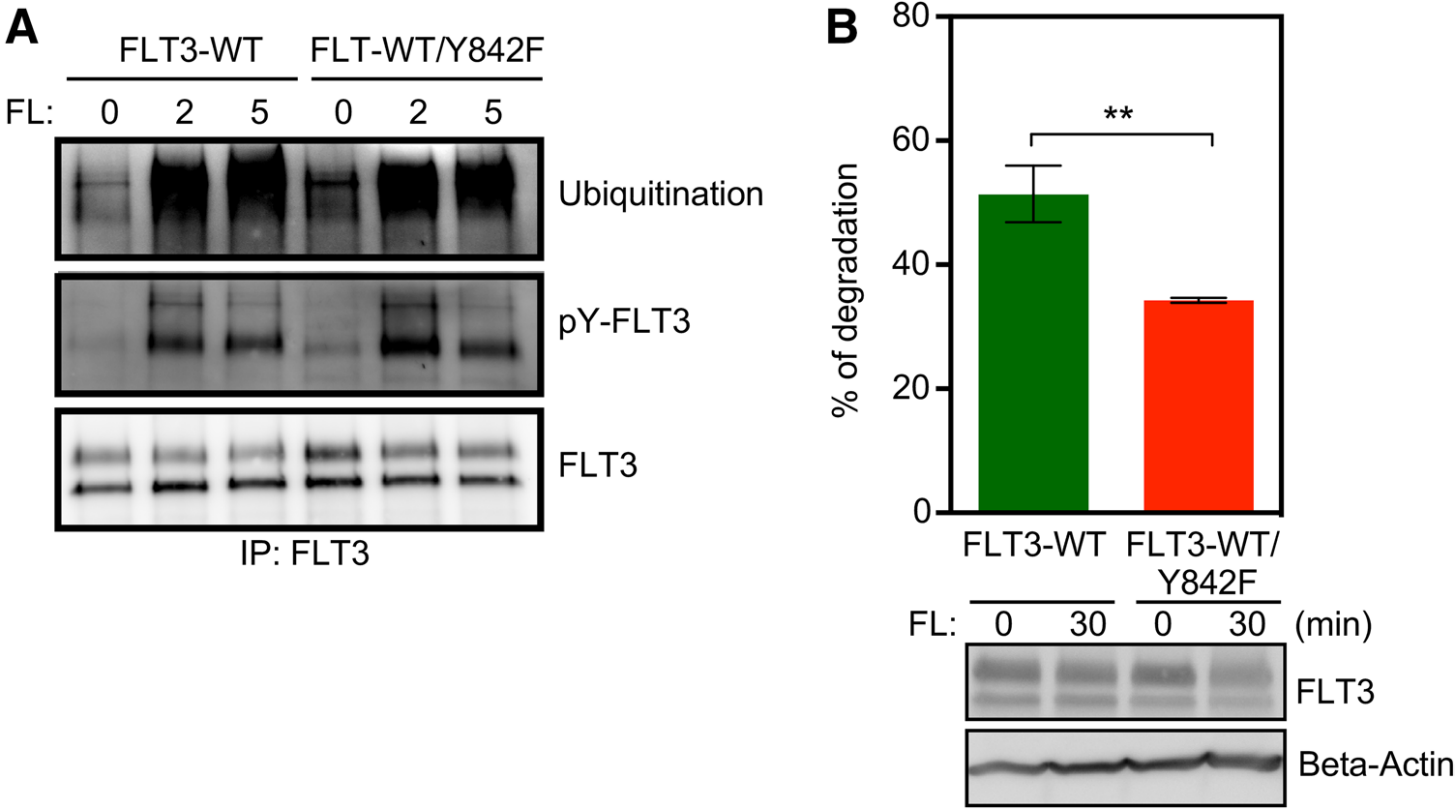
Y842F mutation increases FLT3 degradation. **a** Cells were serum- and cytokine-starved for 4 h before stimulating with 100 ng/ml FL for different time points. Cells were then lysed and lysates were immunoprecipitated using 1 µg anti-FLT3 antibody. **b** Cells were treated with cycloheximide for 30 min followed by 30 min of ligand stimulation. Cells were then lysed and lysates were used to measure degradation. **p < 0.01; *error bar* represents SEM

Ligand stimulation results in dimerization of FLT3 followed by autophosphorylation at several tyrosine residues, which in turn creates docking sites for, among other things, the ubiquitin E3 ligase CBL. In a previous report, we observed that ligand stimulation of wild-type KIT versus ligand stimulation of the activation loop tyrosine mutant of KIT (Y823F) leads to differential tyrosine phosphorylation as well as ubiquitination patterns [16]. In this study, we did not see any significant difference in ligand-induced ubiquitination or tyrosine phosphorylation between wild-type FLT3 and FLT3/Y842F (Fig. 6a). However, FLT3 degradation was increased by approximately 30% following ligand stimulation (Fig. 6b) in the Y824F mutant compared to wild-type FLT3.

## Discussion

Oncogenic mutations in FLT3 lead to aberrant activation of survival and proliferation signaling. The most frequent gain-of-function mutation, FLT3-ITD, is a potent and constitutive activator of downstream signaling. Other gain-of-function mutations include point mutations in the tyrosine kinase domain such as D835Y. Mutation in the tyrosine kinase domain also occurs in combination with a FLT3-ITD mutation leading to resistance to several FLT3 kinase inhibitors. Mutations of the activation loop tyrosine of FLT3 (Y842) is a less frequent event in leukemia. One report suggested that Y842C mutation results in constitutive activation of the receptor [25]. Y842C or Y842H mutations in combination with FLT3-ITD led to resistance to FLT3 kinase inhibitors [26, 27]. In the crystal structure of autoinhibited FLT3, Y842 makes extensive interactions with neighboring amino acid residues and thereby stabilizes the DFG-out inactive form of FLT3 [10, 17]. Mutation of Y842 to either H or C would likely result in loss of these interaction and lead to destabilization of the inactive form of FLT3. In contrast, mutation to F does most likely not interfere with the hydrophobic interactions and thus does not activate FLT3. Interestingly, in the closely related RTK KIT activating mutations have also been described at the homologous tyrosine residue, Y823. Different substitution mutations of this residue have been found but while Y823D is a constitutively active mutant [28], Y823A is kinase inactive [29] and Y823F does not affect kinase activity [16, 29].

To understand the functional role of Y842 we generated an Y842F mutant which is identical to wild-type FLT3 with the exception of the hydroxyl group missing in this position, thus preventing it from being tyrosine phosphorylated. We observed that Y842 is of great importance for FLT3-induced downstream signaling despite the fact that it does not affect the kinase activity of FLT3.

Although phosphorylation of the activation loop tyrosine residues is critical for the kinase activity of several receptor tyrosine kinases such as the fibroblast growth factor receptor, the insulin receptor and MET [12–14], data suggest that it is dispensable for activation of type III RTKs [15–17]. However, Y842 in FLT3 and the corresponding residue in KIT (Y823) play important roles in receptor signaling as well as in transformation mediated by oncogenic mutants of either RTK. We observed that the Y842F mutation significantly reduced FLT3-ITD-induced cell viability and induced apoptosis in transfected 32D cells suggesting that Y842 plays a role in survival signaling mediated by oncogenic FLT3. Furthermore, the Y842F mutant cells exhibited impaired transforming capacity displayed as a reduced capacity to form colonies, which also were smaller, as well as reduced capacity to form tumors in xenografted mice. Thus, Y842F mutation displays in the FLT3-ITD background a similar phenotype to the Y823F mutation in the KIT/D816V background [15], in that it severely limits the oncogenic capacity of the transforming mutant.

Although the Y823F mutation suppresses several signaling pathways downstream of KIT, including AKT, ERK1/2 and p38 [16], the Y842F mutant of FLT3 selectively reduced only FLT3-mediated ERK1/2 signaling, while AKT signaling was intact. This further supports the notion that the receptor activation per se is not affected but selective downstream signaling events. The suppression of ERK1/2 signaling that we observed in mutant cells is likely to be due to the impaired activation of SHP2 signaling. SHP2 is a potent binding partner of FLT3, phosphorylated by FLT3 [30] and required for FLT3-mediated downstream signaling [24]. Activation of SHP2 occurs through binding of its two SH2 domains to phosphotyrosine residues, that leads to a conformational change and activation of its intrinsic protein tyrosine phosphatase activity [29]. Despite being a phosphatase, SHP2 is linked to positive signaling and activation of the RAS/ERK pathway through several mechanisms (for a review, see [10]). The observation that mutation of Y842 significantly reduced SHP2 interaction with FLT3 indicates that the impaired activation of ERK1/2 signaling in Y842F mutant cells is due to the reduced binding of SHP2 to FLT3 and reduced activation. The Y842F mutation did not completely eliminate the binding of SHP2, which can be explained by Y599 being an additional SHP2 binding site [24]. Taken together, our data suggests a unique function of the activation loop tyrosine residue in FLT3. Given the importance of both the activation loop and SHP2 in FLT3-ITD-mediated transformation, the development of drugs that interfere with binding of SHP2 to Y842 or with the activity of SHP2 could be useful drugs for the treatment of patients with acute leukemia.

### Electronic supplementary material

Below is the link to the electronic supplementary material.

Supplementary material 1 (DOCX 220 KB)

## Abbreviations

AML: Acute myeloid leukemia
FBS: Fetal bovine serum
FL: FLT3 ligand
FLT3: Fms-like tyrosine kinase
ITD: Internal tandem duplication
MET: Hepatocyte growth factor receptor
RTK: Receptor tyrosine kinase
SH2: Src homology 2
