# Supplementary material for: Tyrosine 842 in the activation loop is required for full transformation by the oncogenic mutant FLT3-ITD
Source: Cell Mol Life Sci. 2017 Mar 7;74(14):2679–88. doi: 10.1007/s00018-017-2494-0 (PMC5487891; doi:10.1007/s00018-017-2494-0)
Supplement: Supplementary file 1 — Supplementary material 1 (DOCX 220 KB) [file 18_2017_2494_MOESM1_ESM.docx]

**Tyrosine 842 in the activation loop is required for full transformation by the oncogenic mutant FLT3-ITD**

Julhash U. Kazi^1,2^, Rohit A. Chougule^1,2^, Tianfeng Li^3^, Xianwei Su^3,4^, Sausan A. Moharram^1,2^, Kaja Rupar^1,2^, Alissa Marhäll^1,2^, Mohiuddin Gazi^1,2^, Jianmin Sun^1,2,5^, Hui Zhao^3^ and Lars Rönnstrand^1,2*^

**Supplementary figures**

**Figure S1:** Cell surface expression of FLT3-ITD (**A**), FLT3-ITD/Y842F (**B**), FLT3-WT (**C**) and FLT3-WT/Y842F (**D**) in stably transfected 32D cells was analyzed by flow cytometry using PE-conjugated anti-FLT3 (black) or isotype control (red) antibody.


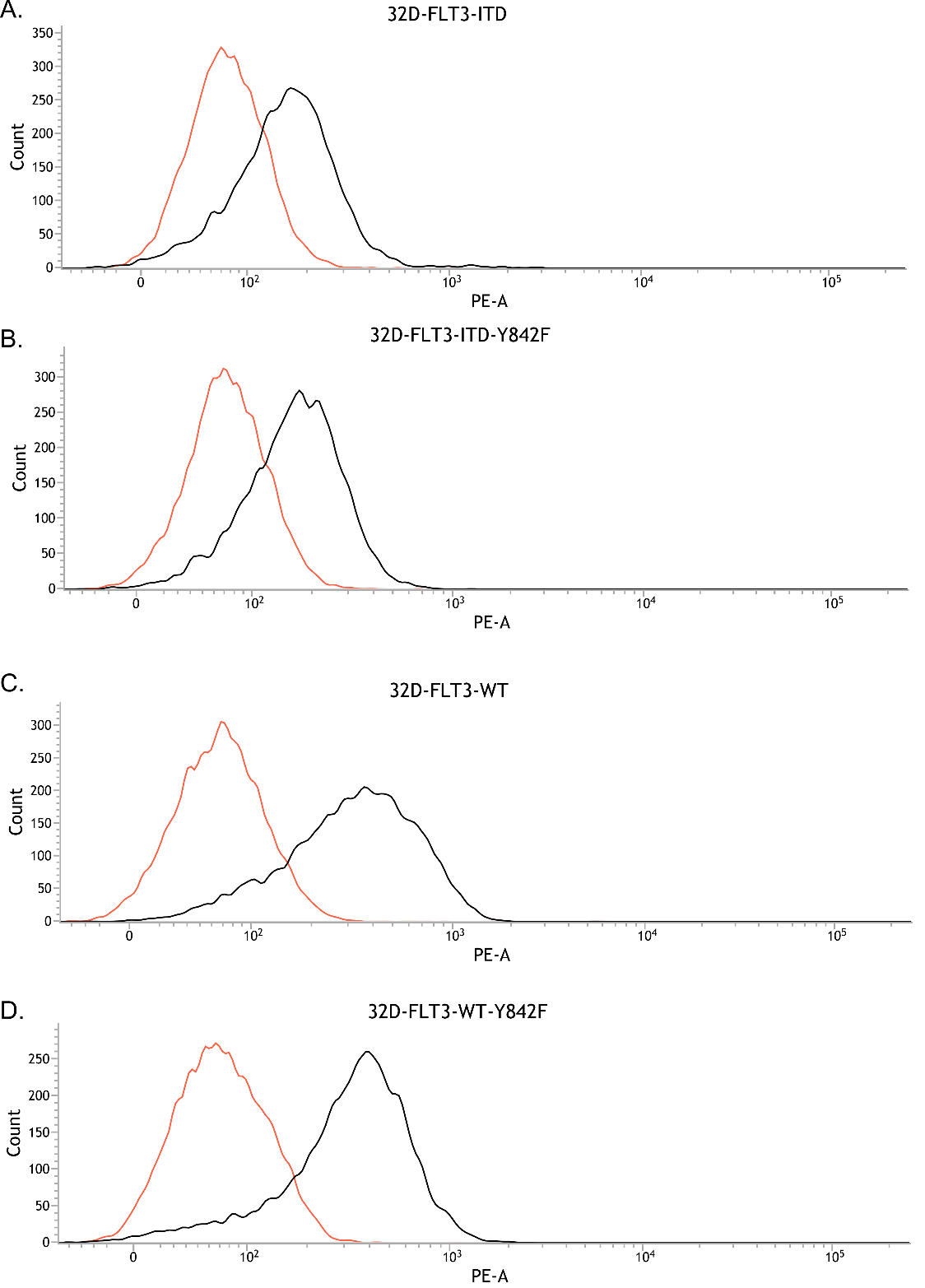


**Figure S2:** STAT5 phosphorylation in FLT3-ITD and FLT3-ITD/Y842F expressing cells: Cells were serum-starved for four hours before lysis. Lysates were used for western blotting analysis.
